# Exploring an Intracellular Allosteric Site of CC-Chemokine Receptor 4 from 3D Models, Probe Simulations, and Mutagenesis

**DOI:** 10.1021/acsptsci.4c00330

**Published:** 2024-07-16

**Authors:** Tianyi Ding, Abdul-Akim Guseinov, Graeme Milligan, Bianca Plouffe, Irina G. Tikhonova

**Affiliations:** † School of Pharmacy, Queen’s University Belfast, Belfast Bt9 7BL, Northern Ireland, U.K.; ‡ Centre for Translational Pharmacology, School of Molecular Biosciences, College of Medical, Veterinary and Life Sciences, University of Glasgow, Glasgow, Scotland G12 8QQ, U.K.; § Wellcome-Wolfson Institute for Experimental Medicine, School of Medicine, Dentistry and Biomedical Sciences, Queen’s University Belfast, Belfast Bt9 7BL, Northern Ireland, U.K.

**Keywords:** CCR4, allosteric site, subtype selectivity, 3D models, cosolvent simulations, mutagenesis, BRET assay

## Abstract

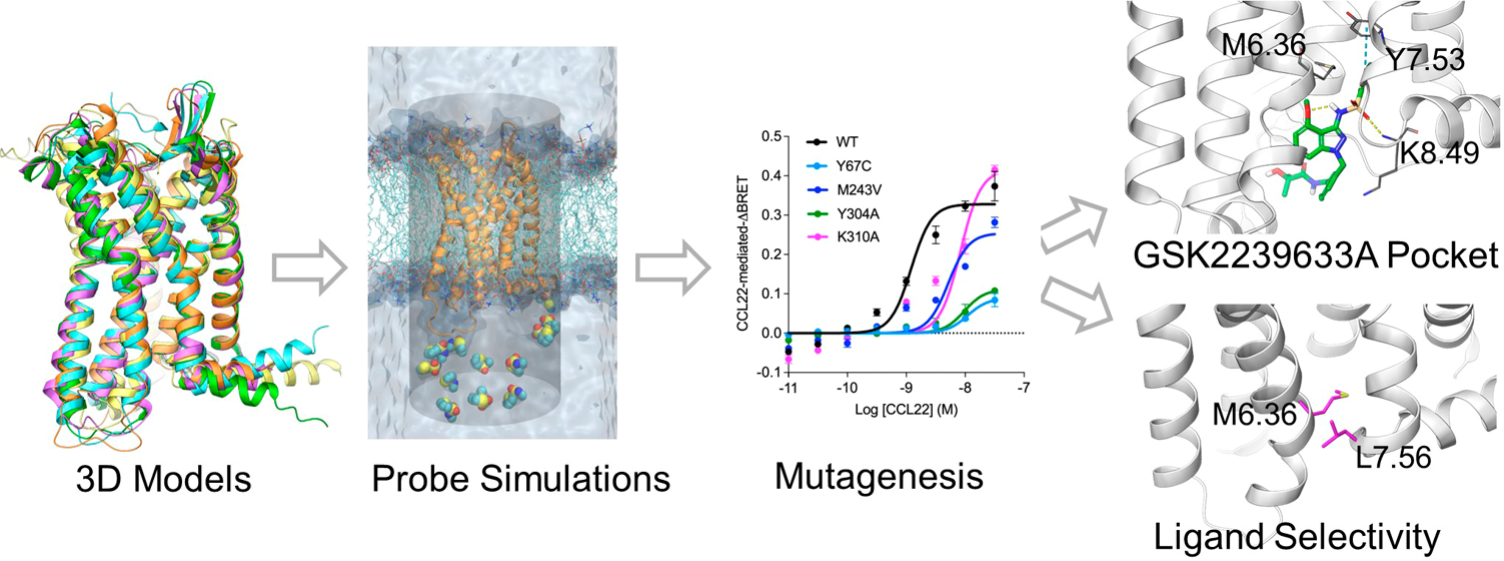

We applied our previously developed probe confined dynamic mapping protocol, which combines enhanced sampling molecular dynamics (MD) simulations and fragment-based approaches, to identify the binding site of GSK2239633A (*N*-[[3-[[3-[(5-chlorothiophen-2-yl)sulfonylamino]-4-methoxyindazol-1-yl]methyl]phenyl]methyl]-2-hydroxy-2-methylpropanamide), a selective CC-chemokine receptor type 4 (CCR4) negative allosteric modulator, using CCR4 homology and AlphaFold models. By comparing the performance across five computational models, we identified conserved (K310^8.49^ and Y304^7.53^) and non-conserved (M243^6.36^) residue hotspots for GSK2239633A binding, which were validated by mutagenesis and bioluminescence resonance energy transfer assay. Further analysis of 3D models and MD simulations highlighted the pair of residues 6.36 and 7.56 that might account for antagonist selectivity among chemokine receptors. Our *in silico* protocol provides a promising approach for characterizing ligand binding sites in membrane proteins, considering receptor dynamics and adaptability and guiding protein template selection for ligand design.

Allosteric sites and their modulators have emerged as promising approaches for drug design in the past 15 years.^1^ They can offer better control of protein function while reducing side effects. Allosteric sites, which can be near or distant from a orthosteric (endogenous ligand binding) site of a protein, often significantly differ within protein classes, allowing selective drug binding. Allosteric modulators, both positive (PAM) and negative (NAM), allow for precise tuning of protein activity by enhancing or impairing orthosteric ligand binding and protein activation.^2,3^

Computational techniques are widely used to identify putative allosteric sites and explore their binding properties.^1,4^ Docking small molecule probes, such as organic solvents or fragments of drug molecules, around static protein structures and scoring their binding pose using energy functions provides a quick assessment of protein pocket properties and possible ligand-binding hotspots.^5−7^ The FTMap online server, which offers efficient and rapid computational solvent mapping, is a popular tool that has been actively used for many drug targets to characterize binding pockets.^8,9^ Cosolvent molecular dynamics (MD) simulations go beyond static structures and allow for the mapping of binding pockets, while considering protein conformational dynamics, water effects, and realistic environment.^10−14^ However, applying this technique to membrane proteins is challenging, as cosolvents partition out of water into the membrane bilayer within nanoseconds, limiting sampling in the protein area of interest during MD simulations.

We have recently developed a probe confined dynamic mapping protocol to identify putative binding pockets in various locations of membrane proteins while considering the membrane environment.^15^ The key feature is the application of a cylinder-shaped harmonic wall potential to limit probe movement to a specified protein region during MD simulations, preventing probe diffusion into the membrane and maintaining its suitable sampling around the protein (Figure 1A). In addition, our protocol benefits from the use of small molecule probes derived from known allosteric modulators. We have successfully tested this approach on G protein-coupled receptors (GPCRs) to locate allosteric sites at extracellular, intracellular, and lipid-interface regions using their X-ray structures.^15^ We reproduced known binding pockets for modulators like LY2119620 (M2 muscarinic PAM),^16^ Cmp-15 (β2 adrenergic NAM),^17^ and BTU (P2Y1 purinergic NAM).^18^ Additionally, our protocol prospectively located the binding site of the D2 receptor PAM UCB compound, which was confirmed by mutagenesis.^15^

**Figure 1:**
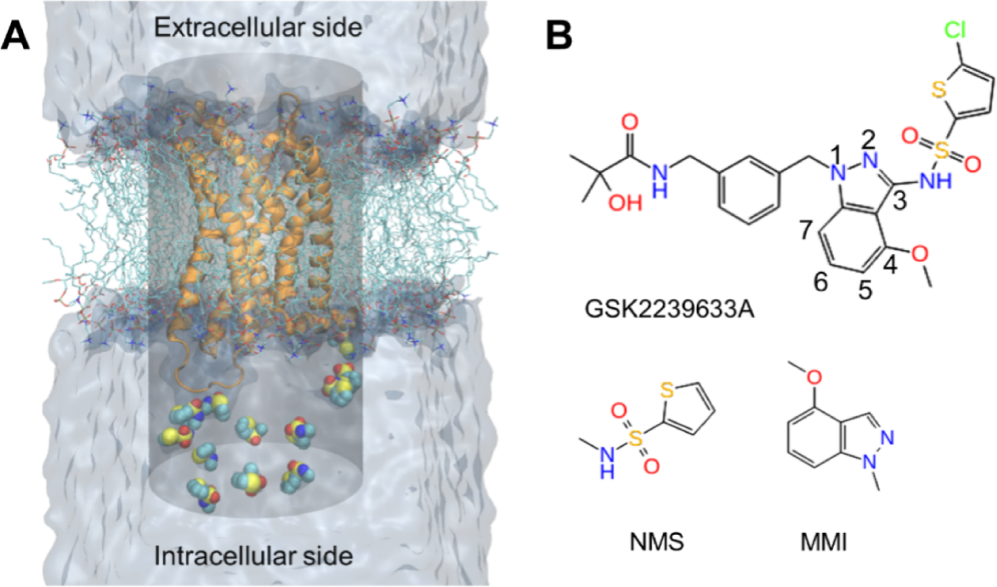
Probe confined dynamic mapping to explore the intracellular pocket of GSK2239633A NAM. (A): Visualization of a receptor (yellow cartoon) in a water−lipid bilayer with probe molecules (space-filling representation) placed at the intracellular side. Water molecules are shown in a surface-like representation, and lipid non-hydrogen atoms are in wire representation. A cylinder-shaped harmonic wall potential is shown as a transparent surface. (B): The intracellular GSK2239633A NAM and probe molecules derived from its structure. The atoms of indazole are labeled.

In this work, we aimed to explore our computational protocol for assessing the performance in GPCR homology and AlphaFold^19^ models. We focus on the chemokine CCR4 receptor, the structure of which remains unsolved. CCR4 is a T-cell-expressed chemokine receptor targeted by C–C motif chemokine ligands CCL17 and CCL22. It is implicated in cancer pathogenesis^20^ and allergic diseases including asthma and dermatitis.^21^ Targeting CCR4 also represents a promising strategy for the treatment of neuropathic pain.^22^ Two chemotypes of selective CCR4 NAMs have been identified by random screening that bind to distinct receptor sites.^23^ The first chemotype includes basic small lipophilic antagonists (e.g., BMS-397 and Z5367428075), which are predicted to bind to the extracellular side of the receptor, partially overlapping with the orthosteric site.^21,24^ The second chemotype includes arylsulfonamide compounds (e.g., AZD2098 and GSK2239633A), which bind to an intracellular site.^24−26^ Despite available modulators, no CCR4-targeted small molecule modulators have been licensed yet, with GSK2239633A failing to meet clinical CCR4 inhibition thresholds.^27^ This highlights the importance of employing structure-based approaches to improve the ligand design outcomes for this receptor.

Here, we used our *in silico* protocol to map the binding site of GSK2239633A^28^ (Figure 1B) at the intracellular side of the receptor using computational 3D models of CCR4. We compared the performance of probe MD simulations across the models, identifying conserved and non-conserved residue hotspots for ligand binding. We next validated four predicted residues through mutagenesis of CCR4 and an enhanced bystander bioluminescence resonance energy transfer (EbBRET) assay.^29^ CCR4 mutants were characterized by using EbBRET biosensors for CCL22-mediated β-arrestin-2 recruitment.

Our integrated computational and experimental approach identified three residues crucial for GSK2239633A-mediated inhibition of CCR4 signaling. We discuss the performance of different receptor computational models in locating allosteric sites and explain the observed selectivity of GSK2239633A relative to other chemokine receptors, demonstrating the utility of our computational tool for enabling allosteric modulator development.

## Results

### Probe Confined Dynamic Mapping in CCR4 Models

To assess the performance of the probe confined dynamic mapping protocol in locating the binding site of GSK2239633A on different CCR4 models, we created homology models of this receptor based on the CCR2, CCR5, CCR7, and CCR9 X-ray structures.^30−33^ The all-residue sequence identity of CCR4 with these receptors ranges from 30% to 46%, while the intracellular binding pocket residues show higher sequence identity: 63%, 72%, 66%, and 57%, respectively (Figure S1). The CCR2, CCR7, and CCR9 structures are resolved with a NAM ligand bound to the intracellular cavity, while the CCR5 NAM binds extracellularly. We also utilized an AlphaFold-predicted model of CCR4.^19^

**Figure 2:**
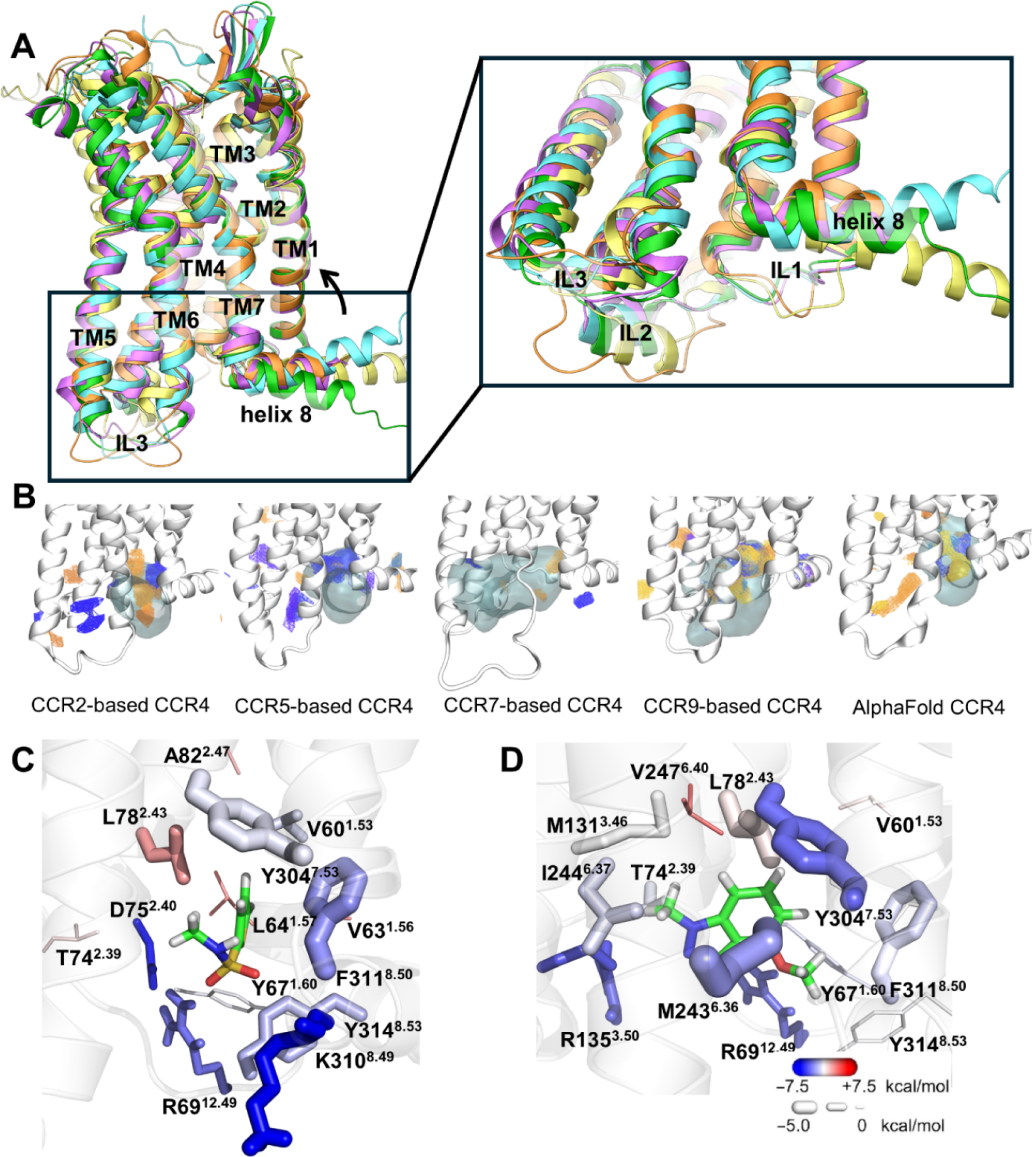
3D CCR4 models and probe molecule binding from MD simulations. (A): Overlay of CCR4 models based on CCR2 (purple), CCR5 (green), CCR7 (orange), and CCR9 (yellow) templates, along with the AlphaFold model (cyan). Transmembrane helices (TM) and intracellular loops (IL) are labeled. The zoomed image on the right shows the intracellular pocket from underneath for clarity, indicated by the black arrow. (B): Overlay of probe density and MDpocket-identified pockets for each CCR4 model. The probe density was calculated using the VMD VolMap tool (isovalue = 0.5). The probe density is in yellow for NMS and in blue for MMI. Binding pockets detected by MDpocket are represented in cyan transparent surface. (C,D): Zoomed views of the binding site on the example of the CCR9-based model of CCR4. Images show a representative snapshot from MD simulations, highlighting key protein−probe molecule interactions. The key residues forming contacts with an NMS or MMI probe molecule are shown in stick representation. The size and color of the residues correspond to the relative strength of van der Waals and electrostatic interactions with the probe, respectively. The actual values of the interaction energies are provided in Table S3.

The overlay of the computational models of CCR4 revealed significant differences at the intracellular side, particularly in the positions of intracellular loops (IL) 1–3, helix 8, and the notably short TM6 in the CCR7-based template (Figure 2A). Conventional MD simulations of the homology models in a lipid bilayer further highlighted variations in the geometrical and physicochemical properties of the CCR4 intracellular cavity across the models. The CCR7- and CCR9-based model cavities exhibited ∼30% larger volume and solvent accessible surface area (SASA) than the CCR2, CCR5, and AlphaFold structures (Table S1). The CCR9 template model displayed the highest hydrophobic and hydrophilic SASA of the intracellular pocket (Table S1). Moreover, MDpocket^34^ analysis revealed variations in the shape of the cavity within the intracellular side of the models (Figure 2B). These observations demonstrate a high variability of the CCR4 intracellular cavity conformations with solvent accessibility being highly dependent on a template choice.

To design the probes for probe-confined dynamic mapping, GSK2239633A was split into two key fragments: *N*-methylthiophene-2-sulfonamide (NMS) and 4-methoxy-1-methylindazole (MMI) (Figure 1B). These fragments represent essential moieties contributing to the compound activity.^25,26,28^ These probes were used to map the intracellular cavity of CCR4 using a cylindrical restraint to maintain the localized sampling of probes near the intracellular side of the receptor (Figure 1A).

The differences in the intracellular cavity shape between the models resulted in varying probe occurrence in a putative binding pocket during the 80 ns MD simulations. NMS occupied the pocket in 7/10 simulations of the AlphaFold CCR4 model, 6/10 in CCR9 and CCR2-based models, 3/10 in the CCR7-based model, and 1/10 in the CCR5-based model. MMI remained in the site in 8/10 simulations of the CCR5-based model, 7/10 simulations of the CCR9-based model, 5/10 simulations of the CCR2-based model, 3/10 simulations of the CCR7-based model, and 2/10 simulations of the AlphaFold-based model. The CCR9-based model had the best probe occurrence for both probes, followed by the CCR2-based model, likely due to the induced fit of the cavity caused by bound NAMs in the template structures. Notably, the CCR2-based model accommodated both probes despite its smaller cavity (Table S1). In the CCR7-based model, the shorter intracellular extension of helix 6 increased the solvent exposure of the pocket, reducing the probe stability (Figure 2A,B). Finally, we observed poor performance of the CCR5-based model with the NMS probe and AlphaFold models with the bulkier MMI probe, which could be explained by the absence of induced fit in these models. Although the frequency of probe occurrence in the intracellular pocket differed considerably among the various models, the probe occupancy exceeded 50%, except for the CCR7 model, whenever the probe entered the pocket (Table S2). Furthermore, all CCR4 models exhibited substantial probe localization to the intracellular pocket near helix 8 (Figure 2B).

The simulations allowed us to detect the NMS/MMI probe−receptor interaction hotspots by assessing the probe–residue interaction energies (Table S3). Figure 2C,D illustrates probe–residue interaction energies using the color and size of binding site residues on the example of the CCR9-based model. Among the conserved residues, D75^2.40^, R69^IL1^, K310^8.49^, and F311^8.50^ (the Ballesteros–Weinstein numbering^35^ is given in superscript) displayed robust electrostatic interactions with the NMS probe in simulations of this model. The interactions with D75^2.40^ and R69^IL1^ were not observed in the other models. Due to different conformations of IL1 in the models, we saw contributions of its various residues to probe binding. K310^8.49^ and F311^8.50^ also showed strong van der Waals interactions (Figure 2C,D, Table S3). Trajectory inspection indicates NMS often forms an initial salt bridge with the K310^8.49^ side chain when entering the pocket and then interacts with the K310^8.49^ backbone once seated while the lysine side chain anchors the ligand from below (Figure S2). This explains the substantial electrostatic and van der Waals energies between the probe and K310^8.49^. The conserved Y304^7.53^ residue also showed significant electrostatic and van der Waals interactions with MMI and van der Waals interactions with NMS in the simulations (Figure 2C,D, Table S3). In the case of non-conserved residues, M243^6.36^ displayed the strongest electrostatic and van der Waals interactions with MMI in all the models apart from the CCR7-based model (Figure 2D). Favorable contacts also occurred with non-conserved Y314^8.53^ and Y67^1.60^, particularly in the CCR5- and CCR9-based CCR4 models.

To validate the binding site of GSK2239633A, we selected conserved K310^8.49^ and Y304^7.53^, and non-conserved M243^6.36^ and Y67^1.60^ for experimental validation.

### Experimental Validation of the CCR4 Allosteric Site

To evaluate the importance of the selected residues, we generated K310^8.49^A, Y304^7.53^A, M243^6.36^V and Y67^1.60^C mutants in CCR4. The non-conserved M243^6.36^ and Y67^1.60^ were mutated to the equivalent residues in CCR2, since GSK2239633A does not inhibit CCL7-mediated CCR2 activation (Figure S3, Table S4).

**Figure 3:**
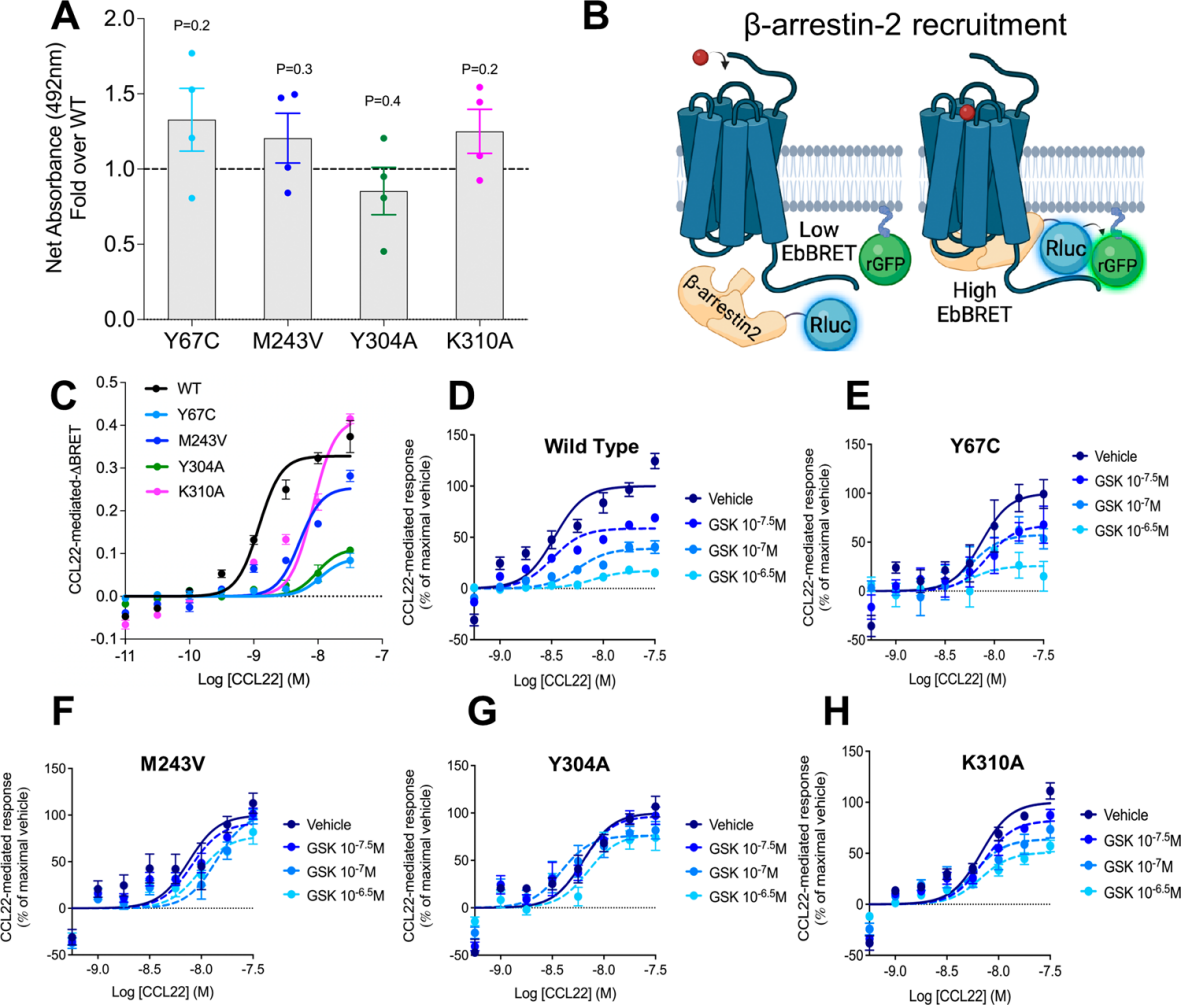
Mutagenesis and β-arrestin-2 recruitment assays demonstrate the importance of K301, Y304, and M243 in the regulation of CCR4 activity by GSK2239633A NAM. (A): Expression of CCR4 mutants at the plasma membrane relative to CCR4 WT measured by ELISA. All mutants are compared with the expression level of wild type set to 1. Each data point represents one experiment performed in triplicates and the mean ± SEM of four independent experiments is represented. No statistical difference was found compared to WT using a one-sample *t* test compared to 1. The *p* values are indicated. (B): Schematic representation of the β-arrestin-2 recruitment EbBRET assay created by BioRender (https://www.biorender.com/). (C): β-arrestin-2 recruitment to the plasma membrane mediated by CCL22 in cells expressing wild-type or mutant CCR4 receptors monitored by EbBRET. Each data point represents the mean ± SEM of four independent experiments performed in triplicates. The concentration–response curve parameters are reported in Table S5. (D–H) Effect of GSK2239633A on CCL22-mediated β-arrestin-2 recruitment at the plasma membrane upon stimulation of wild-type and mutant CCR4 receptors. Data represent the mean ± SEM of four independent experiments performed in triplicates. The concentration–response curve parameters are reported in Table S6.

Before testing the impact of GSK2239633A on the CCR4 mutants, we first verified their cell surface expression in non-permeabilized HEK293T cells by enzyme-linked immunosorbent assay (ELISA) to detect the 3xHA epitope tag sequence fused to the extracellular N-terminus of these receptor constructs (Figure 3A). No significant variation in cell surface expression was observed for any of the mutants compared to that of the wild type receptor. We next evaluated the ability of each mutant receptor to recruit β-arrestin-2 to the plasma membrane upon CCL22 stimulation. The EbBRET biosensor consists of β-arrestin-2 fused to a highly fluorescent mutant of luciferase from *Renilla reniformis* (RlucII)^36^ and green fluorescent protein (rGFP) from the same species fused to the membrane-anchored CAAX motif of K-Ras protein^29^ (Figure 3B). Receptor activation leads to the recruitment of β-arrestin-2 to the plasma membrane, bringing RlucII close to the membrane-anchored rGFP. This proximity enhances bystander resonance energy transfer from RlucII to rGFP, increasing the rGFP fluorescence intensity. Monitoring this change of bystander resonance energy transfer allows us to evaluate the effect of receptor mutations on β-arrestin-2 recruitment compared to the wild-type receptor.

M243^6.36^V and K310^8.49^A exhibited slightly reduced responsiveness to CCL22 (Figure 3C and Table S5), while Y304^7.53^A and Y67^1.60^C showed a more dramatic impact, although some response was still detectable (Figure 3C and Table S5).

Next, we tested the effect of the mutations on the regulation of CCL22-promoted CCR4 activity by GSK2239633A. In the presence of the maximal concentration of GSK2239633A used in the present study (10^–6.5^ M), CCL22-mediated β-arrestin-2 recruitment was only 17.3% ± 1.9% of the level for wild-type CCR4 without the modulator. In contrast, M243^6.36^V, Y304^7.53^A, and K310^8.49^A still recruited β-arrestin-2 at 77.5%, 77.2%, and 51.0% of the levels without GSK2239633A, respectively (Figure 3D,F–H, Table S6). This highlights the important role of M243^6.36^, Y304^7.53^, and K310^8.49^ for responsiveness to GSK2239633A. Unlike the other mutants, Y67^1.60^C did not show any major loss of inhibition by GSK2239633A (Figure 3E, Table S6). Our data confirms that M243^6.36^, Y304^7.53^, and K310^8.49^ are important for functional response to GSK2239633A.

We also tested the effect of the CCR4 mutations on the receptor regulation by Z5367428075, which is predicted to bind to the extracellular side of CCR4. In the presence of Z5367428075, the maximal CCL22-mediated β-arrestin-2 recruitment was only 32.4% ± 3.3% of the level for wild-type CCR4 without the modulator. As expected, the mutants showed only minor changes in Z5367428075 responsiveness for the concentrations tested (Figure S4 and Table S7). Thus, at maximal concentration of Z5367428075 used (10^–5.5^ M), Y67^1.60^C, M243^6.36^V, Y304^7.53^A, and K310^8.49^A recruited β-arrestin-2 at 8.0%, 19.8%, 26.8%, and 34.1% of the levels without the modulator, respectively, similar to wild-type CCR4 (Table S7). Our data confirms that the binding pocket of Z5367428075 does not involve these residues.

### Binding Site of GSK2239633A from Docking and MD Simulations

The validated K310^8.49^, Y304^7.53^, and M243^6.36^ residues were used to define a docking box for docking GSK2239633A to the CCR4 models. The ligand was successfully docked in the CCR9-based model with the default scaling factor for the van der Waals radii of the receptor atoms (Figure S5). In this pose, the sulfonamide group pointed toward the K310^8.49^ backbone, while the aromatic portions contacted Y304^7.53^ and M243^6.36^ residues, as expected. To achieve a similar ligand position in the CCR2, CCR5, CCR7, and AlphaFold-based models, the scaling factor had to be reduced from 1.0 to 0.7. We also docked the ligand to the receptor conformations obtained from probe simulations, where the receptor was bound to either NMS or MMI probe molecules. We observed that the ligand was capable of binding in the expected orientation to these probe-bound receptor conformations by using the default docking protocol (Figure S6).

**Figure 4:**
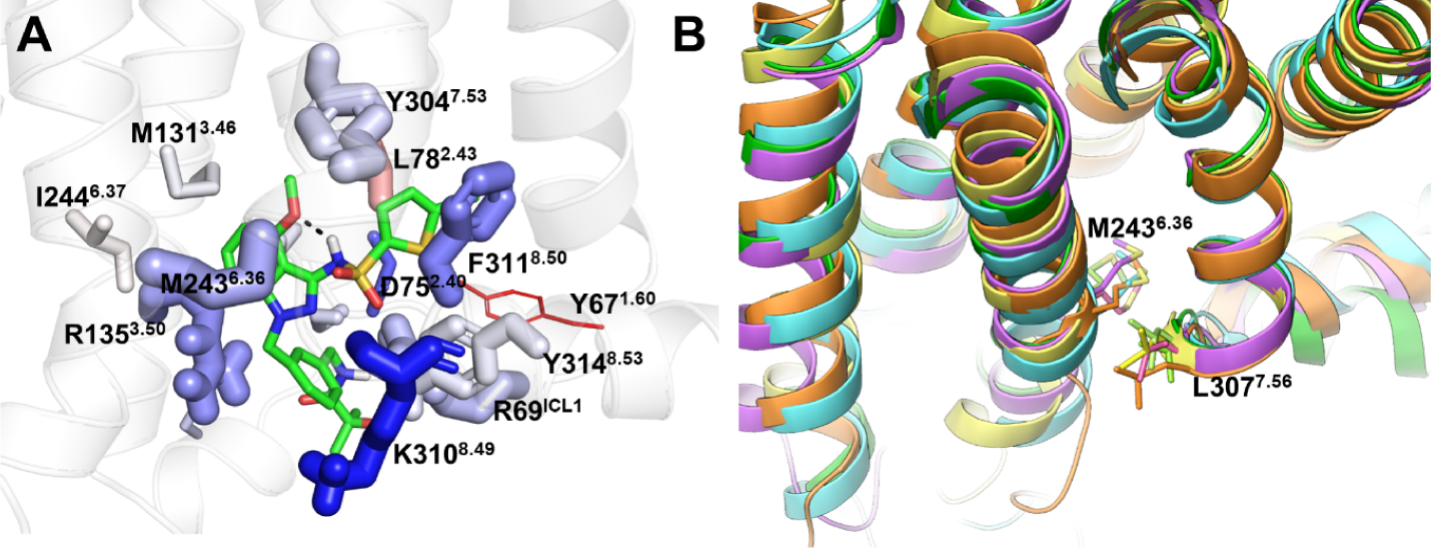
GSK2239633A binding mode and proposed ligand selectivity residues in CCR4. (A): Zoomed views of the binding site. The image shows a representative snapshot from MD simulations, highlighting key protein−ligand interactions. The key residues forming contacts with an NMS or MMI probe molecule are shown in stick representation. The size and color of the residues correspond to the relative strength of van der Waals and electrostatic interactions with the probe, respectively. The actual values of the interaction energies are provided in Table S3. (B): Overlay of CCR4 models based on CCR2 (purple), CCR5 (green), CCR7 (orange), and CCR9 (yellow) templates, along with the AlphaFold model (cyan). M243 and L307 important for selectivity are shown in stick representation.

To further verify the role of key residues, the docked complex of GSK2239633A in the CCR9-based model of CCR4 was subjected to MD simulations. Ligand-residue energy calculations revealed strong electrostatic and van der Waals interactions with K310^8.49^, forming hydrogen bonds with its backbone and the sulfonamide group of the ligand (Figure 4A). Additionally, the side chain of K310^8.49^ engaged in cation−π interactions with the phenyl group of the ligand. Y304^7.53^ also formed significant electrostatic and van der Waals contacts with the ligand through π–π interactions with the thiophene ring.

The non-conserved residue M243^6.36^ exhibited substantial electrostatic and van der Waals interactions with the ligand due to its side chain interaction with the indazole moiety of GSK2239633A (Table S8). We studied the effects of the M243^6.36^V mutation on GSK2239633A binding by simulating the GSK2239633A-M243^6.36^V complex. We observed an increased ligand mobility and altered positioning due to steric hindrance with the valine, as shown by higher root-mean-square fluctuation (RMSF) (3.0 ± 0.8 Å vs 1.6 ± 0.7 Å) values compared to the wild type. The M243^6.36^V mutation introduced repulsive interactions with the ligand and disrupted its interaction with K310^8.49^ (Table S8), supporting the crucial role of M243^6.36^ in stabilizing the GSK2239633A-CCR4 complex, which complements the mutagenesis data.

We analyzed the binding pose of GSK2239633A in the context of available structure–activity relationship (SAR) data.^28^ Hydroxyl analogues at the C4 position exhibit the highest affinity (pIC50 > 7.9).^28^ X-ray structures of these compounds reveal an intramolecular hydrogen bond between the hydroxyl substituents and the sulfonamide group,^26,28^ suggesting that this interaction may be crucial for maintaining the active conformation. This hypothesis is supported by the significantly lower affinity (pIC50 = 5.8)^28^ of a 3-thiophene substituent at C4, which precludes the formation of this intramolecular hydrogen bond. Our MD simulations confirm the presence of this intramolecular hydrogen bond (Figure 4A), demonstrating its importance in maintaining the bioactive conformation by orienting the sulfonyl moiety toward the backbone of E309^8.48^, K310^8.49^ and F311^8.50^ in helix 8. This enables the aromatic moieties to adopt a nearly planar conformation that facilitates aromatic interactions with the receptor. Moreover, hydrophilic substituents at the N1 benzyl group enhance both the potency of GSK2239633A analogues and their solubility.^28^ Our proposed binding pose provides a rationale for this observation as the N1 benzyl moiety interacts with the side chains of K310^8.49^ and R135^3.50^ and is oriented toward the aqueous environment near the intracellular region. We further observed that GSK2239633A binding to helix 8 with its N1 benzyl moiety interacting with R135^3.50^ stabilizes the R135^3.50^–D134^3.49^ ionic lock, a conserved feature of the inactive state in group A GPCRs,^37^ making this interaction present in 30% of the simulation frames as compared to 4% in the absence of the modulator. Thus, NAM activity of GSK2239633A could be attributed to both stabilizing the ionic lock and providing steric hindrance to the receptor–G protein interactions.

## Discussion

The probe confined dynamic mapping protocol effectively characterized the intracellular binding site of GSK2239633A in the CCR4 homology and AlphaFold models. The protocol identified conserved residues K310^8.49^ and Y304^7.53^, as well as the non-conserved residue M243^6.36^, which interacted with the ligand despite variations in model performance. The mutation of the non-conserved Y67^1.60^ located at the start of IL1 and predicted to be important by CCR9- and CCR5-based models did not change receptor responsiveness to GSK2239633A indicating high flexibility of the region and its distinct conformation in CCR4 compared to the CCR9 and CCR5 receptors. Among the tested models, the CCR9-based CCR4 model performed the best, likely due to the presence of a sulfonamide NAM in the CCR9 structure that induced a conformational change in the binding cavity, making it more suitable for accommodating GSK2239633A. The probe confined dynamic mapping protocol outperformed static docking, which failed for four out of five models due to narrow cavities. This further highlights the importance of considering receptor dynamics and binding site adaptability to increase the exposure of hydrophobic pockets, which the probe simulation protocol captures.^14^ The success of the CCR9 template, which had the lowest sequence identity of 57%, compared to the CCR5 template with the highest sequence identity of 72%, suggests that an induced fit is more critical for docking probes or larger ligands than sequence identity. Furthermore, this study demonstrates that receptor conformations obtained from probe simulations with a probe sitting at the expected binding hotspot can accommodate GSK2239633A in docking across all models. Thus, homology modeling of an unknown receptor structure for binding site prediction can be guided by probe density derived from probe simulations to yield a structure capable of accommodating docking probes or larger ligands. In addition, experimental validation of predicted binding site residues using CCR4 mutants reinforces the predictive power of the probe confined dynamic mapping protocol and offers insights into ligand–receptor SAR.

The allosteric site on the intracellular side of the chemokine receptors is highly conserved (Figure S1). A key structural feature of this site is the conserved glycine at position 8.47, located at the start of helix 8. This glycine creates a hinge that exposes the backbone hydrogen bond donors of conserved residues at positions 8.48, 8.49, and 8.50 of helix 8 (E309, K310 and F311 in CCR4). As shown by the probe simulations, these residues, particularly residues at 8.49, serve as the primary anchoring points for NAMs to interact with the receptor (Table S3). Despite the high conservation of the allosteric site, the chemokine receptors exhibit selective binding of NAMs at this location. For instance, GSK2239633A is a selective antagonist with a pIC50 of 7.4 for CCR4 and <5 for other chemokine receptors.^28^ We also show that GSK2239633A does not inhibit CCL-7 mediated CCR2 activation (Figure S3). Our study demonstrates that mutating the non-conserved Met at position 6.36 to Val, as found in CCR2, significantly reduces the potency of GSK2239633A in CCR4. This position varies in hydrophobic residues among the chemokine receptors. The CCR4 models reveal that this residue interacts with the residue at position 7.56, which also varies in the chemokine receptors and is valine in CCR2 (Figure 4B). Our CCR4 and CCR2 MD simulations showed that the intracellular tips of TM6 and TM7 are more rigid in CCR2 compared to those in CCR4 (Table S9). Valines at 6.36 and 7.56 in CCR2 form tighter hydrophobic interactions and reduce the volume of the intracellular cavity (Figure S7) than methionine and leucine in CCR4. This also is likely the reason why, out of ten of NMS and MMI probe simulation trajectories in CCR2, only two have had the NMS probe enter the expected pocket.

Interestingly, chimeric studies have shown that swapping the C-terminus, including residues after the highly conserved NPxxY motif of TM7 and helix 8, between CCR4 and CCR5, alters the selectivity of pyrazinyl-sulfonamides for these receptors.^38^ CCR4-selective antagonists exhibit a 50-fold potency reduction in the chimeric CCR4 with the CCR5 C-terminus, while their potency in the chimeric CCR5 with the CCR4 C-terminus is equivalent to that in wild-type CCR4. Analysis of this highly conserved region in the 3D receptor models identifies only a non-conserved residue at position 7.56 that faces the binding cavity, underscoring its direct role in ligand selectivity.

We, therefore, hypothesize that the hydrophobic interactions between residues at positions 6.36 and 7.56 in the chemokine receptors determine the relative positions of TM6, TM7, and helix 8. This, in turn, influences the accessibility to the backbone of residues at positions 8.48–8.50 and shapes the surrounding binding pocket. Therefore, interactions between residues 6.36 and 7.56 may account for the varied antagonist selectivity among the chemokine receptors.

Intracellular allosteric sites have been identified in several group A GPCRs through X-ray crystallography and cryo-electron microscopy.^39−41^ In the β2 adrenergic receptor (β2AR), NAM Cmpd-15PA binds similarly to NAMs in the chemokine receptors, interacting with TM7 and helix 8, albeit through different residue side chains (PDB: 5X7D).^39^ The neurotensin 1 receptor (NTSR1) NAM SBI-553, which inhibits Gq activation and acts as a β-arrestin-biased allosteric ligand, binds more centrally in the intracellular cavity. It interacts with TM1–3, TM6–7, and helix 8 with one aromatic moiety extending deeper into the helical core (8JPB).^40^ In the free fatty acid 3 receptor (FFA3), PAM AR420626 binds deep within the intracellular cavity, interacting with TM3 and TM5–6 (8J20).^41^ These structures reveal distinct binding modes for allosteric modulators. NAMs typically bind below the conserved D/ERY motif, blocking the R^3.50^–G protein interaction. In contrast, the FFA3 PAM binds above the D/ERY motif, disrupting the R^3.50^–D^3.49^ ionic lock and enabling R^3.50^ to interact with a G protein. These structural insights highlight the diverse mechanisms by which intracellular allosteric modulators can influence GPCR function.

In conclusion, the probe confined dynamic mapping protocol provides a promising approach for characterizing ligand binding sites in GPCRs, accounting for receptor flexibility, guiding template selection for homology modeling, and informing novel ligand design.

## Methods

### 3D Model Building

The homology models of human CCR4 (UniProt D: A0N0Q1) were built based on the X-ray structures of the CCR2 (PDB code: 5T1A), CCR5 (4MBS), CCR7 (6QZH), and CCR9 (5LWE) receptors using the Prime module of Schrödinger software with a default template-based protocol. The AlphaFold2 model of human CCR4, AF-A0N0Q1-F1, was obtained from the AlphaFold2 protein structure database.^19^ The CCR2 structure for simulations was obtained from the PDB code 5T1A.

### Cheminformatics Analysis

The cheminformatics toolkit (frags2img.py, getcore.py, and enumfrags2pdf.py) of OpenEye (OEChem TK 2.2.0)^42^ was employed to conduct ligand fragmentation by functional groups.

### Probe Confined Dynamic MD Simulations

We used the probe simulation protocol from our previous work.^15^ The scripts to run this protocol are available on GitHub (https://github.com/irinat12/Probe-Confined-Dynamic-Mapping-Protocols-GPCRs_membrane_proteins/). The CHARMM36 force field^43,44^ was used for proteins, lipids, and water, while ligand and probe parameters were derived from CgenFF, v 1.0.0.^45^ The receptors were placed in a 90 × 90 Å 1-palmitoyl-2-oleoyl-*sn*-glycero-3-phosphocholine (POPC) membrane patch and solvated with a 30 Å buffer on both sides. The systems were neutralized by 0.15 M Na^+^ and Cl^–^ ions. MD simulations were performed using NAMD Git-2017-12-19, Linux-x86_64-multicore-CUDA.^46^ Three equilibration steps were performed: 1000 steps of minimization followed by 0.5 ns of NPT simulations with the fixed system, 500 minimization steps followed by 2.0 ns of NPT simulations with harmonic restraints on all protein atoms, and 10 ns of NPT with the receptor free to relax with translation on the center of mass removed. The production step included 240 steps of minimization and 80 ns of simulations. Harmonic restraints were applied on the protein Cα atoms with *z* ± 5 Å from the origin, and translation on the protein center of mass was removed. The probe molecules were initially placed randomly within a semiclosed cylindrical region. This cylinder had an open boundary facing the receptor, allowing the probes to interact with the protein surface, while preventing their diffusion into the surrounding environment. All of the simulations were performed at 310 K.

### Standard MD Simulations

GSK2239633A parameters were obtained using Open Force Field.^47^ GSK2239633A was used in the protonated form (p*K*_a_ = 6.60). Membrane-receptor systems were built in the CHARMM-GUI server^48^ with 260 molecules of POPC lipids, and NaCl 0.15 mM. CHARMM-GUI files were converted into NAMD2 format, and simulations were run in NAMD with the CHARMM36 force field. The Langevin thermostat (1.0 ps^–1^ friction coefficient) was used for equilibration and production runs. Equilibration with pressure control and a 1 fs time step was divided into 6 steps, releasing positional restraints sequentially. The protein and ligand were restrained during the whole equilibration time. The scaling factor for the position restraint energy function was decreasing from 10.0 to 5.0, 2.5, 1.0, 0.5, and 0.1 at steps 1, 2, 3, 4, 5, and 6, respectively. A 500 ps NPT simulation (2 fs time step) was the last equilibration stage. Equilibration and production were run in the NPT ensemble with semi-isotropic pressure control using the Monte Carlo barostat. The nonbonded force cutoff was 12 Å. Three 400 ns production runs were started from the same equilibrated structure with the same conditions. All generated replicas were used for pocket cavity property analyses.

### Trajectory Analysis

Probe occupancy was calculated using an *in-house* Tcl script in VMD 1.9.3.^49^ The script counts the presence of any probe molecule within 4 Å around residues at positions 2.43 (X), 7.56 (Y), and 8.48 (Z), which was defined as a center of the intracellular pocket. Occupancy is expressed as the percentage of frames with at least one probe present. The probe density was calculated using the Volmap tool of VMD 1.9.3 with a 1 Å cell side and averaged over all frames of the top molecule. The Volmap probe density was analyzed at isovalues of 0.5. MDpocket 3.0^34^ predicted pockets in all CCR4 3D models. The residue–probe or residue–ligand interaction energy was calculated using the “namdenergy.tcl” script v 1.6 of NAMD^50^ for residues within 5 Å of a probe. Modeling pictures were created with Pymol 2.5.0,^51^ VMD 1.9.3, and Maestro.^52^ The SASA of allosteric binding sites was calculated based on MDpocket-identified pockets using VMD 1.9.3. RSMD and RMSF of the receptor backbone and non-hydrogen atoms of the ligand and hydrogen bonds with the angle cutoff of 90° and the distance of 3.5 Å were calculated with VMD 1.9.3.

### Molecular Docking

The Glide docking program^53^ of Schrödinger software 2019-3 was used for docking of GSK2239633A, prepared using the “Ligand Prep” module. All docking calculations were run in the “Standard Precision” (SP) mode with default parameters. The scaling factor of the van der Waals radii of receptor atoms was set to 1.0 or 0.7. The docking box was set based on K310^8.49^, Y304^7.53^, and M243^6.36^. The best-docked structure was chosen based on GlideScore,^53^ mutagenesis results, and probe simulations.

### DNA Plasmids

All DNA constructs were cloned into the pcDNA3.1(+) expression plasmid except if stated otherwise. rGFP-CAAX,^54^ β-arrestin-2-RlucII,^55^ GFP10-Gγ2,^55^ and Gαi1-loop-RlucII^56^ were previously described. Gβ1, 3xHA-tagged CCR2, and 3xHA-tagged CCR4 were obtained from the cDNA Resource Center of the Bloomsburg University Foundation.

### Site-Directed Mutagenesis

All cDNA templates were cloned into the pcDNA3.1(+) plasmid. Mutations were created using a PCR-based overlapping approach using Phusion High-Fidelity DNA Polymerase (ThermoFisher Scientific). Each reaction (50 μL) contained 200 μM of dNTP mix (Life Technologies), 2 U/uL of Phusion High-Fidelity DNA Polymerase, 1 ng of template DNA, and 0.5 μM of forward and reverse primers in 1× Phusion HF Buffer. A first PCR reaction was performed to generate the megaprimers by combining the forward (F) primer 5′-CCCTATTGACGTCAATGACG-3′ with a mutation specific reverse (R) primer and the R primer 5′-TGCTATTGTCTTCCCAATCC-3′ with a mutation specific F primer:

CCR4-Y67C–F: 5′-CTGTTCAAAT**G**CAAGCGGCTCAGGTCC-3′

CCR4-Y67C-R: 5′-GAGCCGCTTG**C**ATTTGAACAGGACCAG-3′

CCR4-M243 V-F: 5′-GCGGTGAAG**G**TGATCTTTGCCGTGGTG-3′

CCR4-M243 V-R: 5′-GGCAAAGATCA**C**CTTCACCGCCTTGTT-3′

CCR4-Y304A-F: 5′-CCCATCATC**GC**CTTTTTTCTGGGGGAG-3′

CCR4-Y304A-R: 5′-CAGAAAAAAG**GC**GATGATGGGATTAAG-3′

CCR4-Y310A-F: 5′-CTGGGGGAG**GC**ATTTCGCAAGTACATC-3′

CCR4-Y310A-R: 5′-CTTGCGAAAT**GC**CTCCCCCAGAAAAAA-3′

A three-step PCR protocol was used with an initial denaturation at 98 °C for 60 s, followed by 35 amplification cycles. Each amplification cycle consisted of denaturation at 98 °C for 10 s and an annealing at 50 °C for 10 s, followed by an extension at 72 °C for 90 s. To terminate the PCR amplification cycles, a final extension step at 72 °C for 5 min was applied. The PCR products were purified by gel electrophoresis (1% agarose in TBE buffer containing 90 mM Tris, 90 mM boric acid, and 2 mM EDTA) at 100 V using a myGel Mini Electrophoresis System (Accuris Instruments). A QIAEX II Gel Extraction Kit (Qiagen) was used to extract the PCR products from the gel, which are the megaprimers for the second PCR reactions. In this second round of PCR, both forward and reverse megaprimers were combined to the forward primer 5-AAAATGTCGTAACAACTCCG-3′ and the reverse primer 5′-ATGACACCTACTCAGACAAT-3′. The PCR products were purified by gel electrophoresis, and the DNA was extracted from the gel using a QIAEX II Gel Extraction Kit. The template vector and the PCR products were cut with KpnI and XhoI restriction enzymes (New England Biolabs) for 1 h at 37 °C followed by dephosphorylation of the vector by incubating for 30 min at 37 °C with 5 units of Quick CIP (New England Biolabs) followed by a 2 min inactivation at 80 °C. The digested template vector and the cut PCR products were purified by gel electrophoresis, and the corresponding DNA bands were purified again using the QIAEX II Gel Extraction Kit. The dephosphorylated vector and the cut PCR products were combined in an approximately 1:3 ratio and ligated overnight at 16 °C using T4 DNA Ligase (New England Biolabs).

*E. coli* 10-Beta competent cells (New England Biolabs) were transformed with the ligation products and spread on ampicillin-LB agar plates (Luria broth with 1.5% agar, 100 μg/mL ampicillin). One single colony was picked and amplified in ampicillin-LB media in a shaker incubator at 37 °C overnight. The plasmids were purified using PureLink HiPure Plasmid Midiprep Kits (Qiagen). Sequencing was performed by using the Eurofins Genomics Mix2Seq service to confirm the mutagenesis.

### Cell Culture and Transfection

The human embryonic kidney HEK293T clonal cell line (HEK293SL cells) was obtained from Stéphane Laporte (McGill University, Montréal, Québec, Canada) and previously described.^29^ These cells were cultured in Dulbecco’s Modified Eagle Medium (DMEM, Gibco) high glucose supplemented with 10% fetal bovine serum (Gibco) and 100 units per mL penicillin–streptomycin (Gibco), maintained at 37 °C and 5% CO_2_. Cells were passaged every 3–5 days using trypsin–EDTA 0.05% (Gibco) and monthly checked for mycoplasma contamination, which were negative. The DNA to be transfected was combined with salmon sperm DNA (Thermo Fisher Scientific) to obtain a total of 1 μg of DNA per condition. Linear polyethylenimine 25K (PEI; Polysciences) was combined with DNA (3 μg PEI per μg of DNA), vortexed, and incubated 20 min before adding a cell suspension containing 300 000 cells per milliliter (1.2 mL of cells per condition). The appropriate volume of cells containing the DNA was seeded, and cells were incubated for 48 h before assay.

### Bioluminescence Resonance Energy Transfer (BRET) Assays

The cell suspension containing the DNA encoding for the BRET/EbBRET biosensors and receptors (untagged wild-type or mutant CCR4 or 3xHA-tagged wild-type CCR2) was seeded in white 96-well plates (Greiner) at 30 000 cells per well. 48 h post-transfection, cells were washed with DPBS (Life Technologies) and assayed in Tyrode’s buffer (137 mM NaCl, 0.9 mM KCl, 1 mM MgCl_2_, 11.9 mM NaHCO_3_, 3.6 mM NaH_2_PO_4_, 25 mM Hepes, 5.5 mM glucose, 1 mM CaCl_2_, pH 7.4) at 37 °C. GSK2239633A was added in Tyrode’s buffer and incubated for 45 min at 37 °C to reach binding equilibrium prior to adding the agonists (CCL22 or CCL7). Immediately after agonist addition, 2.5 μM of *Renilla* luciferase substrate coelenterazine 400a (DeepBlue C; NanoLight Technology) was added to each well, and the plates were incubated 5 min at 37 °C before BRET/EbBRET measurements. All BRET/EbBRET measurements were performed using a FLUOstar Omega microplate reader (BMG Labtech) with an acceptor filter (515 ± 30 nm) and a donor filter (410 ± 80 nm). BRET/EbBRET values were determined by calculating the ratio of the light intensity emitted by the acceptor over the light intensity emitted by the donor. ΔBRET and ΔEbBRET values are defined as the values of BRET or EbBRET in the presence of agonist minus the value obtained in the absence of agonist (vehicle only). Data normalized as the percentage of maximal vehicle were obtained by setting the maximal ΔEbBRET values in the absence of GSK2239633A as 100%. Concentration–response curves were fitted using nonlinear regression using a 4-parameter equation, and the basal ΔEbBRET was fixed to zero. Statistical significance of parameters of concentration–response curves (agonist-induced maximal efficacy or potency) was established by comparing independent fits with a global fit that shares the selected parameter using the extra sum-of-squares *F* test. Data are expressed as mean ± standard error of the mean (SEM), and *n* represents the number of independent experimental repeats. GraphPad Prism 9.4.1 was used to plot data.

### ELISA

To measure the relative cell surface expression of the mutant and wild-type CCR4 receptors (all tagged with 3xHA epitopes at their amino-terminal), the cell suspension containing the DNA was seeded in white 96-well plates previously coated with Poly-d-Lysine (Bio-Techne) at 30 000 cells/well (100 μL per well). For the coating, the Poly-d-Lysine solution (0.1 mg per ml) was added (50 μL per well) and the plates were incubated at 37 °C for at least 30 min. Following the incubation, the solution was aspirated and wells were washed two times with DPBS before adding the cell suspension containing DNA. 48 h after seeding, cells were washed with DPBS and fixed by adding 50 μL per well of 4% paraformaldehyde in PBS and incubated at room temperature for 10 min. The fixing solution was aspirated, and wells were washed 3 times in a washing buffer composed of DPBS containing 0.5% BSA (Merck Life Science). The washing buffer was left in the wells for 10 min following the last wash. After the 10 min incubation, the buffer was removed, 50 μL per well of monoclonal 3F10 anti-HA-peroxidase (Merck Life Science) 12.5 ng/mL in washing buffer was added, and the plate was incubated for 1 h at room temperature. The antibody was aspirated, and wells were washed 3 times with the washing buffer. The washing buffer was left in the wells for 10 min following the last wash, and wells were washed again 3 times with DBPS only. After aspiration of the DPBS, 100 μL per well of SigmaFast OPD (Merck Life Science) solution prepared as recommended by the manufacturer was added. Wells were incubated in the presence of the OPD solution until the wells containing cells expressing receptors become yellow (typically 10 min). The reaction was stopped by the addition of 25 μL per well of hydrochloride 3 M in water. 100 μL per well was transferred to a transparent clear 96-well flat bottom plate (Thermo Fisher Scientific), and the absorbance at 492 nm was measured using a FLUOstar Omega microplate reader. The net absorbance (absorbance measured in the presence of receptor minus the absorbance measured in the absence of receptor) was calculated and normalized to the net absorbance of the wild-type CCR4 receptor set to 1.

## Supporting Information Available

MD simulations, pharmacology experiments, and amino acid sequence alignment (PDF)
PDB files from docking and probe simulations used to create figures (ZIP)
