# Supplementary material for: Exploring an Intracellular Allosteric Site of CC-Chemokine Receptor 4 from 3D Models, Probe Simulations, and Mutagenesis
Source: ACS Pharmacol Transl Sci. 2024 Jul 16;7(8):2516–26. doi: 10.1021/acsptsci.4c00330 (PMC11320731; doi:10.1021/acsptsci.4c00330)
Supplement: Supplementary file 1 — pt4c00330_si_001.pdf [file pt4c00330_si_001.pdf]

Supporting Information

Exploring an intracellular allosteric site of CC-chemokine receptor 4 from 3D models, probe simulations and mutagenesis

Tianyi Ding[1], Abdul-Akim Guseinov[1], Graeme Milligan[3], Bianca Plouffe[2*] and Irina G. Tikhonova[1*]

[1]School of Pharmacy, Queen's University Belfast, Belfast, Northern Ireland, UK

[2]Wellcome-Wolfson Institute for Experimental Medicine, School of Medicine, Dentistry and Biomedical Sciences, Queen's University Belfast, Belfast, Northern Ireland, UK

[3]Centre for Translational Pharmacology, School of Molecular Biosciences, College of Medical, Veterinary and Life Sciences, University of Glasgow, Glasgow, Scotland, UK.

Table S1: Intracellular pocket properties in the 3D CCR4 models during standard MD simulations. Volume of the cavities were assessed by the MDpocket program. $SASA_{phil}$, and $SASA_{phob}$ are solvent-accessible surface area for the hydrophilic and hydrophobic residues for the MDpocket identified cavity.

Run No.	Volume, Å^3	$SASA_{all}$, Å^2	$SASA_{phob}$, Å^2	$SASA_{phil}$, Å^2
**CCR4 with the CCR2 template**				
**1**	740±138	2130±108	653±62	1465±93
**2**	718±158	2122±130	736±61	1359±97
**3**	790±150	2276±90	840±45	1377±69
**overall**	750±152	2176±131	743±95	1400±99
**CCR4 with the CCR5 template**				
**1**	693±172	2403±96	832±66	1570±66
**2**	632±196	2427±117	849±70	1578±72
**3**	496±134	2083±113	645±49	1438±83
**overall**	607±188	2304±191	775±111	1529±98
**CCR4 with the CCR7 template**				
**1**	1087±195	3385±138	1601±105	1703±68
**2**	1031±173	2935±141	1332±94	1525±79
**3**	957±150	3290±180	1284±93	1702±116
**overall**	1025±182	3203±248	1406±170	1643±123
**CCR4 with the CCR9 template**				
**1**	685±143	3666±181	1624±93	2042±119
**2**	1167±214	3765±108	1718±72	2048±64
**3**	892±174	3784±112	1639±72	2145±71
**overall**	915±267	3739±147	1660±90	2078±100
**CCR4 with the AlphaFold template**				
**1**	752±204	2197±167	921±146	1159±86
**2**	732±205	2094±157	819±114	1154±62
**3**	544±148	2034±174	787±120	1135±78
**overall**	676±210	2108±179	843±140	1149±77

Table S2. Probe occupancy in the last 40 ns of MD simulations. The pocket area selection for probe occupancy calculation was based on the MDpocket and Volmap druggable site prediction and defined as XYZ coordinates: -12 < X < 2, -2 < Y < 9, -25 < Z < -10.

Probe	Template	Occupancy (%)	Number of trajectories
**NMS**	CCR2	52±33	6
**NMS**	CCR5	62±0	1
**NMS**	CCR7	97±3	3
**NMS**	CCR9	76±34	6
**NMS**	Alphafold	85±21	7
**MMI**	CCR2	69±38	5
**MMI**	CCR5	97±9	8
**MMI**	CCR7	20±17	3
**MMI**	CCR9	97±7	7
**MMI**	Alphafold	100±0	2

Table S3. The average probe-residue interaction energy (Etot), involving van der Waals (Evdw) and electrostatic (Eele) components from probe simulations. The measurements were taken only in the frames when a probe molecule sits in the putative binding site.

Average MMI-residue interaction energy in CCR4 (kcal/mol)

CCR2 template				CCR5 template				CCR7 template			
Residue	Eele	Evdw	Etot	Residue	Eele	Evdw	Etot	Residue	Eele	Evdw	Etot
**Y304**	-1.33	-2.71	-4.04	**F311**	-1.85	-4.26	-6.11	D75	-17.32	-1.45	-18.77
**F311**	-1.44	-2.37	-3.80	**Y67**	-0.09	-5.46	-5.55	**F311**	-1.92	-1.92	-3.84
**M243**	-0.44	-2.70	-3.14	**K310**	-1.32	-3.46	-4.79	**M243**	-0.61	-1.68	-2.29
L78	0.13	-2.51	-2.38	**Y304**	-1.22	-2.91	-4.14	G308	-0.89	-1.10	-1.99
K68	-1.33	-0.87	-2.20	R135	-2.26	-1.03	-3.29	D134	-1.62	-0.12	-1.74
**K310**	-1.63	-0.38	-2.01	**M243**	-0.65	-2.30	-2.95	**K310**	2.18	-3.49	-1.30
I244	-0.16	-0.97	-1.13	V64	0.05	-2.16	-2.11	L78	0.14	-1.37	-1.23
T74	-0.04	-1.04	-1.08	I244	-0.26	-1.19	-1.45	E309	-0.58	-0.65	-1.23
M131	0.21	-1.26	-1.05	Y314	0.58	-1.94	-1.37	R135	1.12	-2.31	-1.19

CCR9 template				AlphaFold model			
Residue	Eele	Evdw	Etot	Residue	Eele	Evdw	Etot
R135	-4.38	-2.32	-6.70	**K310**	-5.08	-1.89	-6.97
**M243**	-1.66	-4.60	-6.26	D75	-4.91	-0.59	-5.50
**Y304**	-2.28	-2.72	-5.00	**F311**	-1.28	-2.42	-3.70
I244	-0.75	-1.68	-2.43	G308	-0.96	-1.88	-2.84
**F311**	-0.70	-1.60	-2.30	**M243**	-0.12	-1.99	-2.11
R69	-1.36	-0.27	-1.63	K68	1.45	-3.31	-1.86
T74	-0.38	-1.20	-1.58	Y314	-0.24	-1.60	-1.84
**K310**	-0.94	-0.63	-1.57	E309	0.45	-1.82	-1.38
M131	-0.08	-1.29	-1.37	V63	-0.29	-0.68	-0.96
L78	0.36	-1.72	-1.36	V64	0.44	-1.20	-0.77

Average NMS-residue interaction energy in CCR4 (kcal/mol)

CCR2 template				CCR5 template				CCR7 template			
Residue	Eele	Evdw	Etot	Residue	Eele	Evdw	Etoi	Residue	Eele	Evdw	Etot
**K310**	-1.48	-0.45	-1.94	K68	-4.79	-0.25	-5.04	**K310**	-8.59	-1.96	-10.55
**F311**	-0.48	-1.12	-1.60	**Y304**	-0.48	-1.72	-2.20	**F311**	-3.23	-3.33	-6.56
**Y304**	-0.24	-1.09	-1.32	**M243**	-0.34	-1.82	-2.17	G308	-2.74	-1.08	-3.82
V60	-0.63	-0.57	-1.20	A240	-0.70	-0.68	-1.38	**M243**	-0.61	-1.59	-2.20
K68	-0.10	-0.96	-1.06	G308	-0.62	-0.43	-1.05	Y314	-0.68	-1.30	-1.98
Y314	-0.40	-0.54	-0.94	I244	-0.03	-0.99	-1.02	A240	-1.05	-0.14	-1.18
V63	0.35	-0.82	-0.46	L78	0.18	-0.56	-0.38	L64	0.17	-1.32	-1.15
L78	0.10	-0.55	-0.44	F311	-0.30	-0.06	-0.35				

CCR9 template				AlphaFold model			
Residue	Eele	Evdw	Etot	Residue	Eele	Evdw	Etot
**K310**	-3.45	-1.41	-4.86	K68	-7.10	-1.43	-8.52
R69	-2.38	-0.83	-3.21	**F311**	-2.74	-3.33	-6.06
**F311**	-1.91	-0.81	-2.71	**K310**	-4.78	-0.70	-5.48
**D75**	-1.74	-0.19	-1.93	**Y304**	-0.24	-3.15	-3.39

**Y304**	-0.09	-0.65	-0.74	**D75**	-1.94	-0.46	-2.41
S72	-0.25	-0.13	-0.38	G308	-1.69	-0.14	-1.83
**M243**	0.35	-0.57	-0.22	Y314	-0.72	-1.09	-1.80
**Y67**	-0.08	-0.13	-0.21	V60	-0.45	-1.17	-1.62
Y314	0.15	-0.34	-0.19	L78	0.44	-1.95	-1.50
V64	0.00	-0.09	-0.09	**M243**	0.26	-1.30	-1.05
L78	0.16	-0.15	0.01	V64	0.54	-1.55	-1.01

Table S4. Effect of the allosteric modulator GSK2239633A on Gαi1 protein activation by CCR2 upon stimulation with CCL7. The potency (pEC_{50}) and maximal efficacy (E_{max}) for each concentration-response curve in Figure S3 are indicated and compared to different GSK2239633A concentrations by comparing independent fits using the Extra sum-of-squares F test. Each data point represents the mean ± SEM of four independent experiments performed in triplicates. *p < 0.05.

Activity	GSK2239633A Concentrations							
	**0M**	**$10^{-7.5}$ M**	**10^{-7} M**	**$10^{-6.5}$ M**	**10^{-6} M**	**$10^{-5.5}$ M**	**10^{-5} M**	**$10^{-4.5}$ M**
**pEC_{50}**	-8.4 ± 0.10	-8.3 ± 0.19	-8.6 ± 0.13	-8.5 ± 0.13	-7.8 ± 0.20*	-8.4±0.25	-8.2±0.23	-8.1±0.20
**E_{max}**	0.14 ± 0.01	0.12±0.01	0.12±0.01*	0.12±0.01	0.14±0.02	0.11±0.02	0.13±0.02	0.14±0.02

Table S5. Parameters of concentration-response curves of β-arrestin-2 recruitment to plasma membrane upon stimulation of wild-type and mutant CCR4 receptors with increasing concentrations of CCL22. The potency (pEC_{50}) and maximal efficacy (E_{max}) for each dose-response curve in Figure 3C are indicated and compared to WT by comparing independent fits using the Extra sum-of-squares F test. Data represents the mean ± SEM of four independent experiments performed in triplicates. *p < 0.05, ***p < 0.001, ****p < 0.0001.

Activity	Mutants				
	**WT**	**Y67C**	**M243V**	**Y304A**	**K310A**
**pEC_{50}**	-8.92 ± 0.05	-7.96 ± 0.07*	-8.29 ± 0.07****	-8.02 ± 0.08***	-8.08 ± 0.06****
**E_{max}**	0.33 ± 0.01	0.09 ± 0.01*	0.25 ± 0.02	0.11 ± 0.01***	0.41 ± 0.03

Table S6. Parameters from the CCL22 concentration-response curves of β-arrestin-2 recruitment at plasma membrane by wild-type and mutant CCR4 receptors in the presence of GSK2239633A. The potency (pEC_{50}) and maximal efficacy (E_{max}) for each concentration-response curve in Figure 3D-H are indicated and compared to CCR4 WT at the same GSK2239633A concentration by comparing independent fits using the Extra sum-of-squares F test. Data represents the mean ± SEM of four independent experiments performed in triplicates. *p < 0.05, **p < 0.01, ***p < 0.001, ****p < 0.0001.

System	Activity	WT	Y67C	M243V	Y304A	K310A
**CCL22**	pEC_{50}	-8.5 ± 0.1	-8.1 ± 0.1	-8.1 ± 0.1	-8.2 ± 0.1	-8.2 ± 0.1
	E_{max} (%)	100 ± 6.1	100 ± 11.9	100 ± 13.8	100 ± 10.4	100 ± 7.9
**CCL22 + GSK2239633A ($10^{-7.5}$ M)**	pEC_{50}	-8.5 ± 0.04	-8.1 ± 0.1**	-8.1 ± 0.1**	-8.2 ± 0.1**	-8.2 ± 0.1**
	E_{max} (%)	58.7 ± 2.7	67.1 ± 11.2	91.9 ± 9.2***	96.7 ± 8.8****	82.1 ± 6.9**
**CCL22 + GSK2239633A (10^{-7} M)**	pEC_{50}	-8.3 ± 0.1	-8.2 ± 0.1	-7.9 ± 0.1**	-8.2 ± 0.1	-8.2 ± 0.1
	E_{max} (%)	39.0 ± 2.6	57.4 ± 8.7*	102.6 ± 15.6****	76.2 ± 7.4****	63.8 ± 7.1**
**CCL22 + GSK2239633A ($10^{-6.5}$ M)**	pEC_{50}	-8.1 ± 0.08	-8.2 ± 0.03	-8.0 ± 0.1	-8.4 ± 0.1	-8.2 ± 0.1
	E_{max} (%)	17.3 ± 1.9	25.7 ± 9.6	77.5 ± 10.2***	77.2 ± 7.7***	51.0 ± 4.0****

Table S7. Parameters from the CCL22 concentration-response curves of β-arrestin-2 recruitment at plasma membrane by wild-type and mutant CCR4 receptors in presence of Z5367428075. The potency (pEC_{50}) and maximal efficacy (E_{max}) for each concentration-response curve in Figure S4 are indicated and compared to CCR4 WT at the same Z5367428075 concentration by comparing independent fits using the Extra sum-of-squares F test. Data represents the mean ± SEM of five independent experiments performed in triplicates. *p < 0.05, **p < 0.01, ***p < 0.001, ****p < 0.0001

System	Activity	WT	Y67C	M243V	Y304A	K310A
**CCL22**	pEC_{50}	-8.3 ± 0.1	-8.3 ± 0.1	-8.1 ± 0.1	-8.1 ± 0.1	-8.3 ± 0.1
	E_{max} (%)	100.0 ± 6.4	100.0 ± 12.0	100.0 ± 7.9	100.0 ± 11.3	100.0 ± 7.8
**CCL22 + Z5367428075 ($10^{-6.5}$M)**	pEC_{50}	-8.1 ± 0.1	-8.5 ± 0.3	-7.9 ± 0.1	-7.9 ± 0.1	-7.9 ± 0.1
	E_{max} (%)	76.2 ± 5.0	29.3 ± 10.4***	55.3 ± 4.4*	59.9 ± 8.9	70.7 ± 6.2
**CCL22 + Z5367428075 (10^{-6}M)**	pEC_{50}	-8.2 ± 0.1	-8.0 ± 0.2	-8.0 ± 0.1	-8.0 ± 0.1	-8.1 ± 0.1
	E_{max} (%)	45.7 ± 4.7	42.7 ± 13.3	34.4 ± 5.2	38.0 ± 7.9	43.5 ± 4.7
**CCL22 + Z5367428075 ($10^{-5.5}$M)**	pEC_{50}	-8.0 ± 0.1	-7.8 ± 2.3	-8.0 ± 0.1	-7.9 ± 0.2	-8.0 ± 0.1
	E_{max} (%)	32.4 ± 3.3	8.0 ± 34.4	19.8 ± 3.4*	26.8 ± 10.1	34.1 ± 6.7

Table S8. The average GSK2239633-residue interaction energy (Etot), involving van der Waals (Evdw) and electrostatic (Eele) components from standard simulations of the wild type and M243V CCR9-based CCR4.

Average ligand-residue wild type CCR9-based CCR4 Energy (kcal/mol)				Average ligand-residue M243V CCR9-based CCR4 Energy (kcal/mol)			
Residue	Eele	Evdw	Etot	Residue	Eele	Evdw	Etot
**K310**	-7.1	-3.5	-10.6	D75	-20.6	-1.8	-22.3
**M243**	-2.6	-5.2	-7.7	F311	0.5	-4.8	-4.3
R135	-3.4	-4.1	-7.5	Y304	-1.9	-2.4	-4.3
F311	-3.8	-3.4	-7.2	M131	-0.9	-1.4	-2.3
R69	-1.9	-3.8	-5.8	T74	1.0	-3.2	-2.2
D75	-4.9	-0.9	-5.8	L146	0.0	-1.7	-1.8
**Y304**	-1.3	-4.0	-5.3	S72	-0.3	-1.3	-1.7
Y314	-0.7	-2.7	-3.4	L70	-0.7	-0.3	-1.1
L78	0.4	-2.9	-2.5	L78	2.0	-2.9	-1.0
S72	-1.0	-1.4	-2.4	Y314	-0.1	-0.9	-1.0
T74	-0.6	-1.6	-2.2	L64	0.1	-0.9	-0.8
S145	-1.5	-0.6	-2.1	L307	0.2	-0.8	-0.5
M131	-0.3	-1.3	-1.5	V63	-0.2	-0.2	-0.4
I244	0.0	-1.5	-1.4	G308	0.8	-1.1	-0.3
L146	-0.4	-0.4	-0.9	Y67	0.8	-0.8	0.0
L70	0.4	-1.2	-0.9	V243	2.7	-2.6	0.1

Table S9. Root mean square fluctuation (RMSF) of the intracellular tips of TM6 and TM7 for CCR2 and CCR4 from the MD simulations.

Systems	RMSF TM6, Å	RMSF TM7, Å
CCR2		
Run1	0.5±0.1	0.7±0.3
Run2	0.5±0.2	0.8±0.3
Run3	0.6±0.2	0.8±0.4
CCR2-based CCR4		
Run1	0.7±0.2	0.9±0.3
Run2	0.8±0.3	1.3±0.4
Run3	0.8±0.2	1.0±0.3

Figure S1: Alignment of residues forming the intracellular binding pocket of CCR4 and chemokine receptors with available experimental structures. The initial all-residue sequence alignment was obtained from the GPCRdb website (https://gpcrdb.org/). Residues facing the putative intracellular binding pocket of the receptor were selected in the GeneDoc program. All sequence identity calculations were performed using GeneDoc.

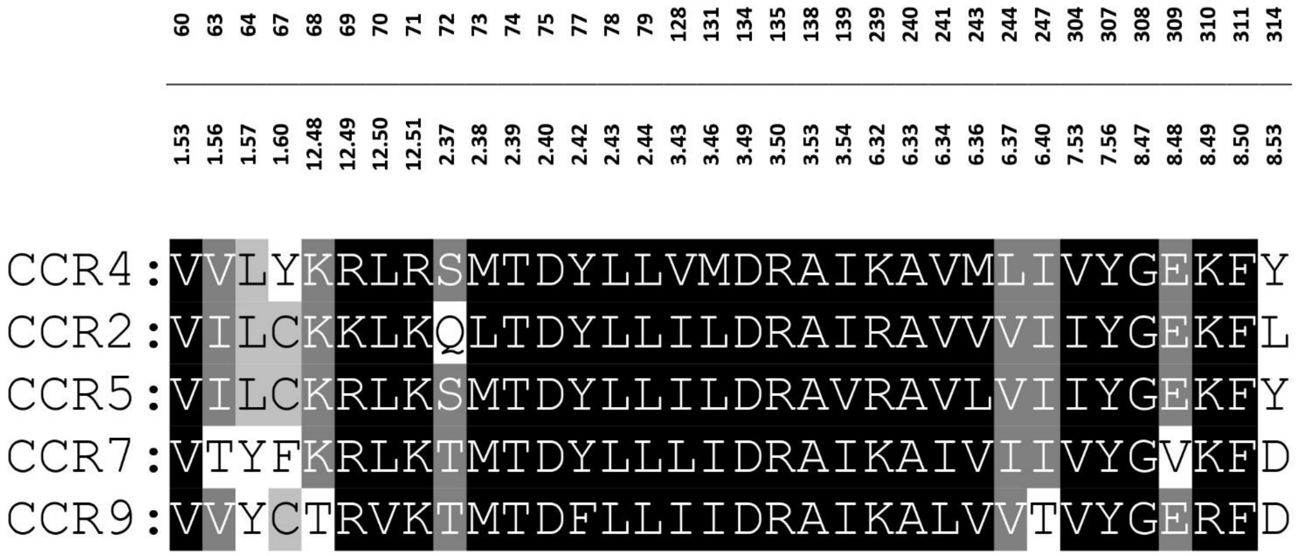

Figure S2: Probe MD simulation snapshots highlighting interactions with K310$^{8.49}$, M243$^{6.36}$ and Y304$^{7.53}$ A and B: The entrance and pocket interactions of NMS with the sidechain and backbone of K310$^{8.49}$. **C:** The interaction of MMI with M243$^{6.36}$.

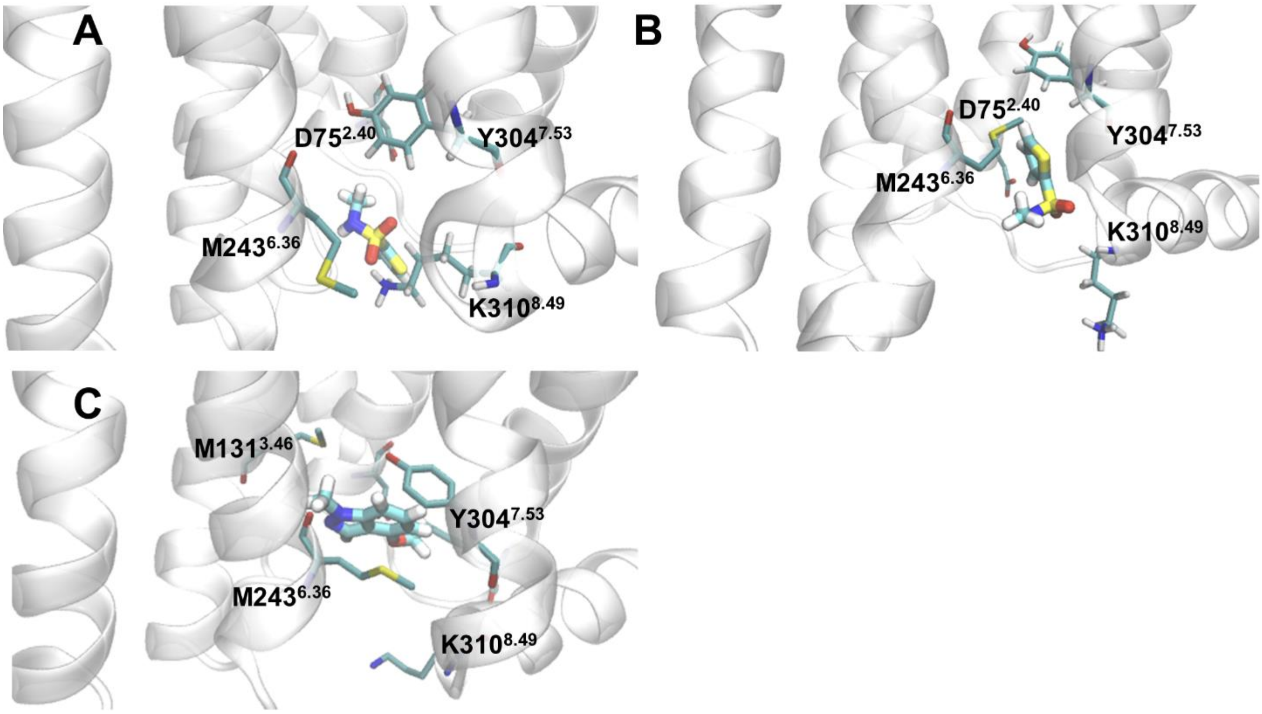

Figure S3: Effect of the allosteric modulator GSK2239633A on Gαi1 protein activation by CCR2 upon stimulation with CCL7. Each data point represents the mean ± SEM of four independent experiments performed in triplicates. The concentration-response curves parameters are reported in Table S4.

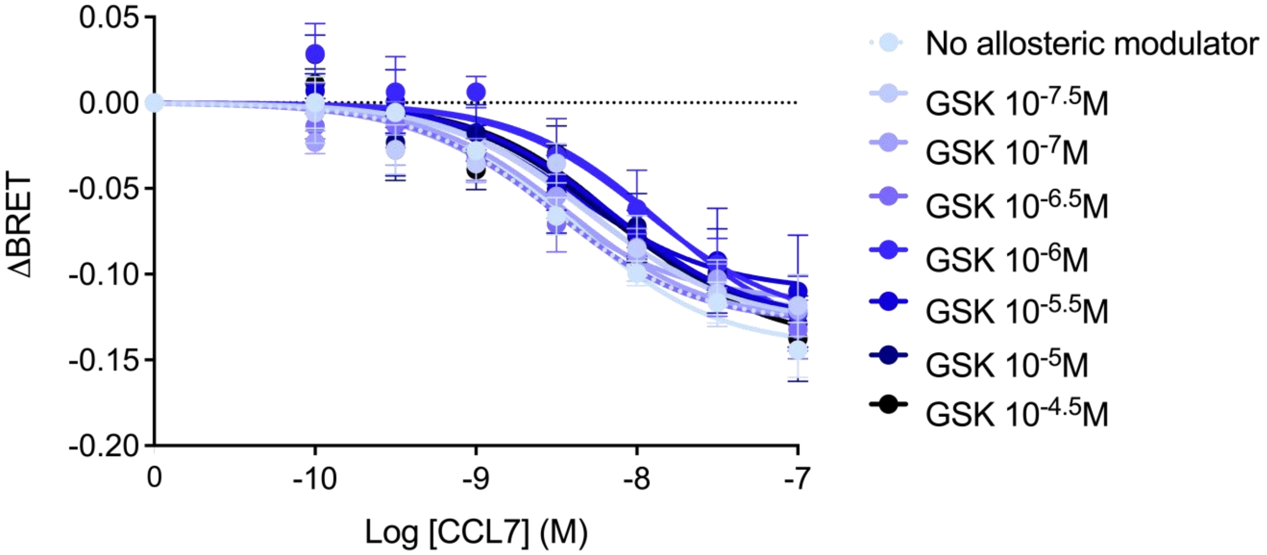

Figure S4: Effect of Z5367428075 on CCL22-mediated β-arrestin-2 recruitment at plasma membrane upon stimulation of wild-type and mutant CCR4 receptors. CCL22 concentration-response curve of β-arrestin-2 recruitment in the absence and presence of increasing concentrations of Z5367428075 (**A**) at wild-type (**B**) or Y67C (**C**), M243V (**D**), Y304A (**E**) and K310A (**F**). Each data point represents the mean ± SEM of at least five independent experiments performed in triplicates. The dose-response curves parameters are reported in Table S7.

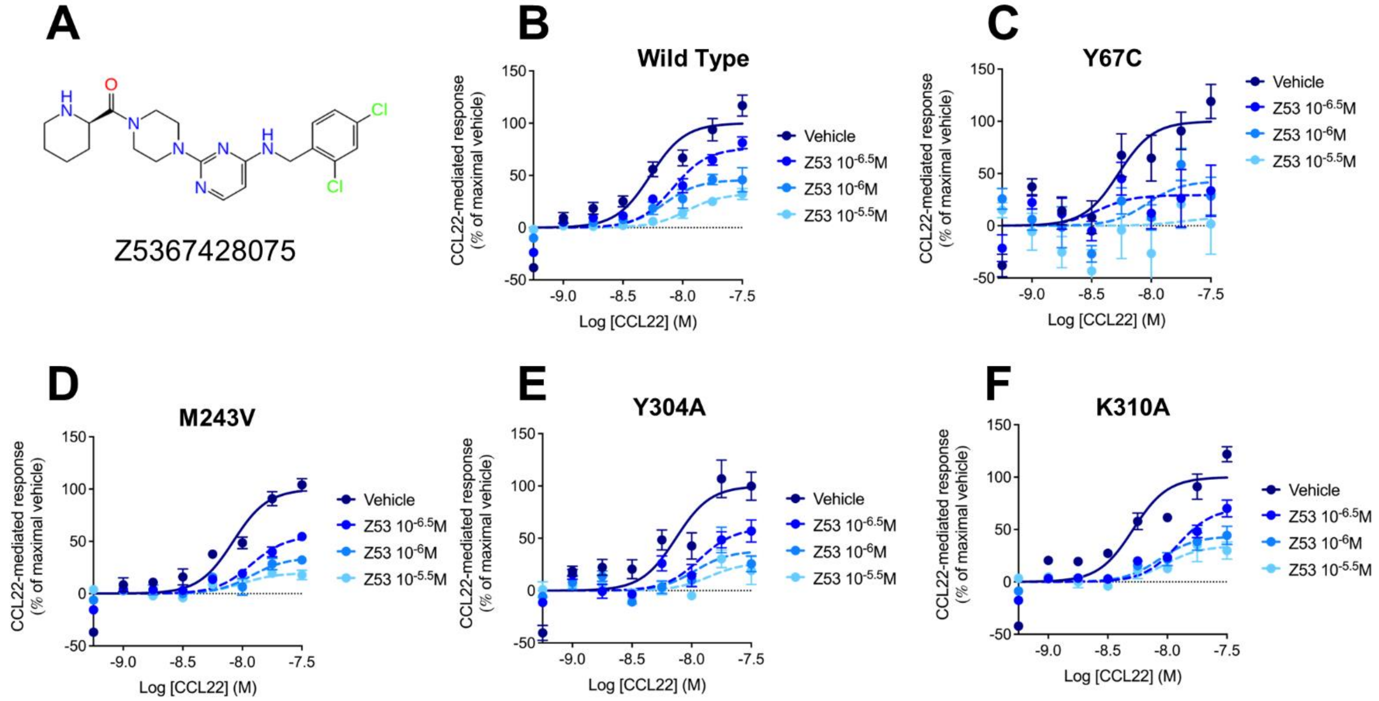

Figure S5: Docking of GSK2239633A to the homology and AlphaFold CCR4 models with the scaling factor for Van der Waals radii of receptor atoms 1.0 and 0.7. Hydrogen bonds and π-π interactions are shown as pink and cyan dashed lines, respectively.

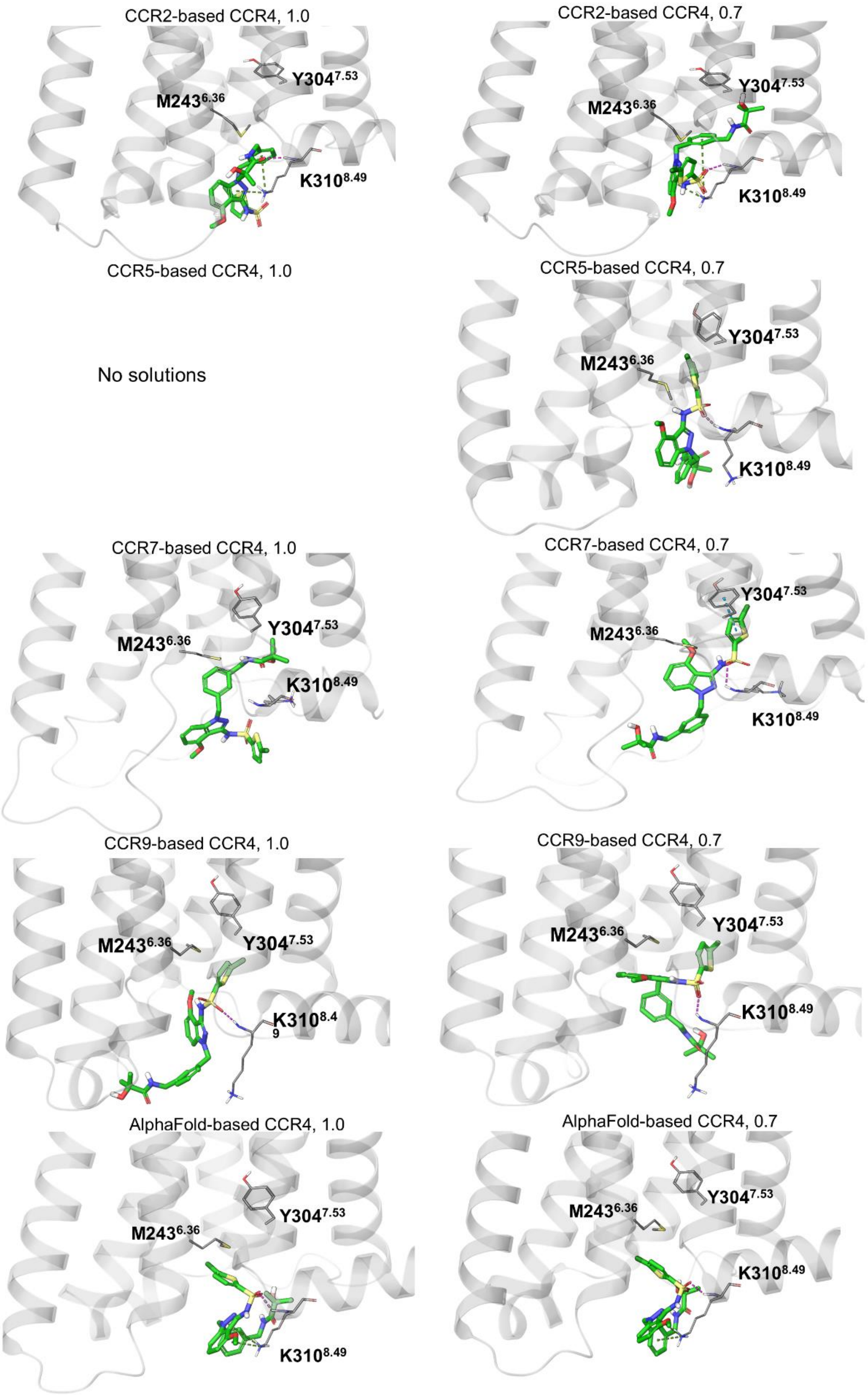

Figure S6: Docking of GSK2239633A to the receptor conformations obtained from probe MD simulations. Docking was completed with the scaling factor for Van der Waals radii of receptor atoms as default, 1.0. Hydrogen bonds and π-π interactions are shown as pink and cyan dashed lines, respectively.

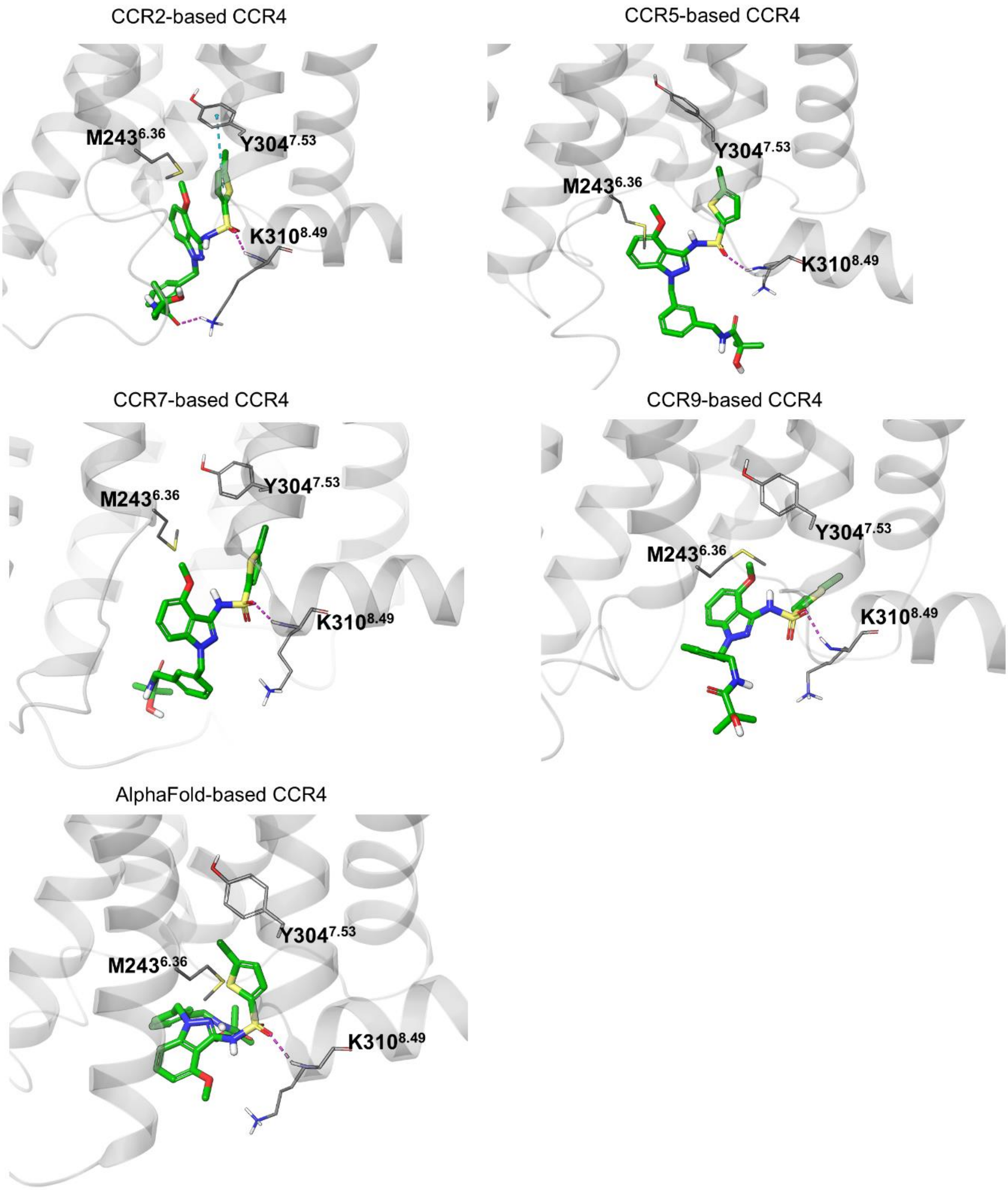

Figure S7: The intracellular cavity in CCR2 identified by MDpocket and its volume.

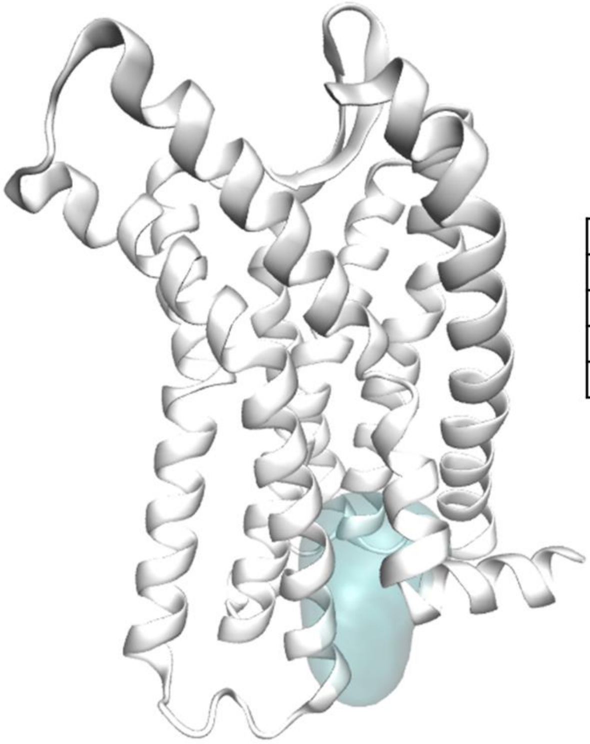

MD simulation	Volume, Å^3
CCR2-Run1	540±101
CCR2-Run2	718±114
CCR2-Run3	626±102
Average	628±128
